# Vast (but avoidable) underestimation of global biodiversity

**DOI:** 10.1371/journal.pbio.3001192

**Published:** 2021-08-12

**Authors:** John J. Wiens

**Affiliations:** Department of Ecology and Evolutionary Biology, University of Arizona, Tucson, Arizona, United States of America

## Abstract

The number of species on Earth is highly uncertain. A recent study has suggested that there are less than 2 million prokaryotic species on Earth; this Formal Comment suggests instead that there are more likely hundreds of millions or billions of species, and that the majority of these are bacteria associated with insects and other animals.

The number of species on Earth is a fundamental number in science. Yet, estimates of global biodiversity have been highly uncertain. There are presently approximately 1.9 million described species [1]. Estimates of the actual number (both described and undescribed) have ranged from the low millions into the trillions [2,3]. Furthermore, described species richness [1] is dominated by animals (1.3 million; 68%), not bacteria (approximately 10,000 species; 0.5%). Larsen and colleagues [2] summarized evidence suggesting that the majority of species on Earth may be bacteria associated with insect hosts and that bacterial richness may push global biodiversity into the hundreds of millions of species or even low billions.

Louca and colleagues [4] (LEA hereafter) have claimed instead that there are only 40,100 host-associated bacterial species among all animal species and 0.8 to 1.6 million prokaryotic species overall (see their “Author summary”). Strangely, they excluded bacterial species associated with animal hosts from their estimates of total prokaryotic diversity and justified this by claiming that the estimates of Larsen and colleagues [2] were “mathematically flawed.” Here, I examine their claims and present new estimates of global biodiversity.

Remarkably, all projections by LEA for host-associated bacterial richness were based on an estimate from one ant genus (*Cephalotes*), an estimate that is demonstrably incorrect by orders of magnitude (S1 Text). Without examining the underlying data [5], LEA estimated only 40 bacterial species among all 130 ant species in this genus. Yet, simply counting the bacterial species among the 25 sampled ant species in that genus reveals 616 unique bacterial species, of which 539 appear to be unique to the genus and 369 each unique to a single ant species (using the standard 97% cutoff for 16S divergence and data from [5]). Thus, there were >500 bacterial species among 25 ant species, not 40 bacterial species among 130 ant species. This mistake was further exacerbated by inexplicably ignoring data from the other 2 insect genera analyzed by Larsen and colleagues [2], thus maximizing the impact of their incorrect estimate for this genus.

Their overall estimate of bacterial richness was also strongly influenced by their questionable assumption that all animal genera can share bacterial species (i.e., reducing their estimate of 3 million host-associated bacterial species to only 40,100). They assumed “a conservative overlap of only 0.1% between any two randomly chosen genera” for the number of bacterial species shared between animal genera. No justification was given for this value of 0.1%, nor were any alternative values explored. Furthermore, they implicitly assumed that any bacterial species can be shared between any pair of animal genera, regardless of their phylogeny, habitat, or geographic range. So, for example, a bacterial species that is a gut endosymbiont of a terrestrial herbivorous insect species endemic to Madagascar could somehow be shared with a deep-sea worm in the northern Pacific Ocean. This is ridiculous: there must be a reason why bacterial species are shared among host species and genera (e.g., shared phylogeny, location, diet). For example, broad-scale studies show that sharing of bacteria among insect hosts is associated with both host phylogeny and diet [6].

LEA stated “it is known that substantial overlap exists between the microbiota of different host genera and even of distantly related animal taxa.” However, they provided no numbers to justify this “substantial overlap.” In fact, none of the papers they cited as supporting this assumption actually do (S2 Text). For example, one study [7] found 5 bacterial species shared among 5 insect genera utilizing the same type of host plant (cycads). However, LEA do not mention that this study found 1,789 unique bacterial species among just these 5 insect species (or 177 after filtering). This seems inconsistent with their estimate of only 40,100 bacterial species across all animals. In summary, rather than estimating the overlap of bacterial species among host genera, LEA simply made a number up and combined this with unrealistic, unsupported assumptions about overlap. If LEA had considered *Cephalotes* (which all their estimates were based on), a survey of this genus and related genera [5] found 1,019 bacterial species, with only 77 of the 616 bacterial species in *Cephalotes* shared with other sampled genera, and the sharing of bacterial species among hosts strongly related to host phylogeny.

Numerous surveys of bacterial diversity in insects strongly suggest that there are far more than 40,100 bacterial species among all animals (Table 1). These studies show that even modest samples of insect species (13 to 31) each reveal >1,000 unique bacterial species (not 40 among 130 host species). Moreover, bacterial richness does not strongly level off as more insects are sampled. Thus, the analysis of 62 insect species [8] found roughly twice as many bacterial species as those of approximately 30 insect species [5,9], and the study of 218 insect species [6] found >3.5 times as many as the study of 62 insect species. The simple fact that a study found 9,301 bacterial species among only 218 sampled insect species strongly suggests that there are more than 40,100 bacteria among all animals.

**Table 1:** Surveys of bacterial diversity among insect species. LEA incorrectly estimated that a genus of 130 ant species (*Cephalotes*) hosts only 40 bacterial species and subsequently assumed that all animal genera have the same low number of bacterial species. These broad surveys of bacterial species among insects suggest that many insects (including *Cephalotes*) host much larger numbers of bacterial species.

Insect group sampled	Insect species sampled	Unique bacterial species found	References
Ants (*Cephalotes* and 3 related genera)	29	1,019	Sanders and colleagues [5]
Lycaenid butterflies	31	1,156	Whitaker and colleagues [9]
Native Hawaiian insects (beetles, flies, true bugs)	13	1,094	Poff and colleagues [10]
Various insect orders	62	2,073	Colman and colleagues [8]
21 insect orders	218	9,301	Yun and colleagues [6]

Given these problems with the estimate of LEA, what is the actual number of bacterial species on Earth? LEA were correct that Larsen and colleagues [2] only estimated the number of species-specific bacteria per insect host species, and those estimates could be wrong. I therefore recalculated those estimates based on more direct counts of species-specific bacteria from the original studies (S3 Text). In Table 2, I present estimates of global biodiversity based on these new estimates. These new estimates are generally within the range of values estimated by Larsen and colleagues [2]. Specifically, Larsen and colleagues [2] projected 0.209 to 5.8 billion species on Earth, of which 66% to 91% are bacteria, whereas I project 0.183 to 4.2 billion, with 58% to 88% bacteria (Table 2).

**Table 2:** Modified projections of global species richness across major groups of organisms. These projections are based on refined estimates of host-specific bacterial richness (details in S3 Text). The 4 scenarios follow Larsen and colleagues [2] and are explained below. For each scenario, the projected number of species for each group is shown, along with the percentage of the total number of species belonging to that group (note that plants are <0.5% and are rounded down to 0%). In addition to the 4 scenarios, 4 other assumptions were explored. The first 3 involve different estimated numbers of morphologically cryptic arthropod species per morphology-based insect species (from 6 to 2 to 0; for justification, see [2]). These impact the number of animal species, and all downstream estimates for other groups. The final, fourth set of analyses assumes 6 morphologically cryptic arthropod species and that mites host negligible numbers of nematode species. Scenario 1 assumes that all animal species have a full set of bacterial, protist, and fungal endosymbionts, even if they are parasites, but that microsporidian fungi and apicomplexan protists have little or no host-specific bacterial richness. Scenario 2 assumes that symbionts have limited numbers of symbionts themselves (i.e., nematodes have an average of only one host-specific bacterial species) and that microsporidians and apicomplexans have few or no bacterial species. Scenario 3 assumes that all animal species have a full set of symbiont species and that microsporidians and apicomplexans host (on average) as many bacterial species as animal species do. Scenario 4 is identical to Scenario 1, except that it assumes that mites have reduced species richness relative to other arthropods (0.25 mites∶1 other arthropod species). Note that there is an error in Table 3, Scenario 1 in Larsen and colleagues [2]: There should be 27.2 million animal species, not 20.4. The correct number is used here. Archaean species is considered to be limited overall [2], and so is not treated separately.

	Scenario 1		Scenario 2		Scenario 3		Scenario 4	
	Million species	% of total	Million species	% of total	Million species	% of total	Million species	% of total
6 cryptic arthropod species								
Animals	163.2	9.4	163.2	13.7	163.2	3.9	102.0	9.4
Plants	0.3	0	0.3	0	0.3	0	0.3	0
Fungi	165.6	9.6	165.6	13.9	165.6	3.9	104.6	9.6
Protists	163.2	9.4	163.2	13.7	163.2	3.9	102.0	9.4
Bacteria	1,240.3	71.6	701.8	58.8	3,721.0	88.3	775.2	71.5
Total	1,732.7		1,194.1		4,213.3		1,084.1	
2 cryptic arthropod species								
Animals	54.4	9.4	54.4	13.6	54.4	3.9	34.0	9.4
Plants	0.3	0	0.3	0	0.3	0	0.3	0
Fungi	56.8	9.8	56.8	14.2	56.8	4.0	36.4	10.0
Protists	54.4	9.4	54.4	13.6	54.4	3.9	34.0	9.4
Bacteria	413.4	71.4	233.9	58.5	1,240.3	88.2	258.4	71.1
Total	579.4		399.9		1,406.3		363.1	
0 cryptic arthropod species								
Animals	27.2	9.3	27.2	13.5	27.2	3.9	17.0	9.3
Plants	0.3	0	0.3	0	0.3	0	0.3	0
Fungi	29.6	10.2	29.6	14.7	29.6	4.2	19.4	10.6
Protists	27.2	9.3	27.2	13.5	27.2	3.9	17.0	9.3
Bacteria	206.7	71.0	117.0	58.1	620.2	88.0	129.2	70.6
Total	291.1		201.3		704.5		182.9	
Mites host limited nematode richness, 6 cryptic arthropod species								
Animals	122.4	9.4	122.4	11.9	122.4	3.9	91.8	9.4
Plants	0.3	0	0.3	0	0.3	0	0.3	0
Fungi	124.8	9.6	124.8	12.1	124.8	3.9	94.2	9.6
Protists	122.4	9.4	122.4	11.9	122.4	3.9	91.8	9.4
Bacteria	930.2	71.5	661.0	64.1	2,790.7	88.3	697.7	71.5
Total	1,300.2		1,030.9		3,160.7		975.8	

In summary, the conclusions of LEA are based on an initial estimate of bacterial richness for one genus that was clearly incorrect, combined with a made-up number (and unrealistic assumptions) to estimate overlap of bacterial species among host genera. Reanalyses here suggest that bacterial richness (and the diversity of life) is more likely in the hundreds of millions or billions.

## Supporting information

S1 Text Estimating bacterial richness among turtle ant species. (DOC)

S2 Text Overlap of bacterial species among host animal genera. (DOC)

S3 Text Refining the estimates of Larsen and colleagues (2017). (DOC)
