# Supplementary material for: Vast (but avoidable) underestimation of global biodiversity
Source: PLoS Biol. 2021 Aug 12;19(8):e3001192. doi: 10.1371/journal.pbio.3001192 (PMC8360379; doi:10.1371/journal.pbio.3001192)
Supplement: S1 Text — (DOC) [file pbio.3001192.s001.doc]

**S1 Text. Estimating bacterial richness among turtle ant species**

Larsen et al. [1] estimated the number of unique bacterial species per insect host species by examining the distribution of bacterial species among closely related insect species. They focused on closely related species given that these species should share the most bacterial species in common (based on previous studies showing greater overlap of bacteria among more closely related host species [2,3]). Thus, these closely related species should provide the most accurate and conservative estimates of host-specific bacterial richness. Larsen et al. [1] found relevant data in the literature on three genera from two of the largest insect orders (Diptera: fruit flies: Drosophila; Hymenoptera: turtle ants: Cephalotes: wasps: Nasonia). Larsen et al. [1] also reviewed broad-scale estimates of host-associated bacterial diversity across insects [2,3]. They found that overall bacterial richness in other insect species (and other insect orders) was generally similar to that of the insect species in which host-specific bacterial numbers were estimated. Thus, there was little evidence that most other insect species have bacterial diversity that is radically different from these three genera.

Larsen et al. [1] performed pairwise comparisons among closely related insect species, identified the number of bacterial species that were shared between each species pair, and assumed that the non-shared species were unique to a given species. They then presented an average of these estimated unique species among pairwise comparisons as the mean number of unique bacterial species per insect host. They estimated that there were 13.4 unique bacterial species per insect host species in Drosophila, 9.6 bacterial species per host species in Nasonia, and 9 in Cephalotes. This latter value for Cephalotes was the mean from two small clades of closely related species within that genus, the 4 ant species of the laminatus clade (with 10 bacterial species per host species) and the 3 ant species of the depressus clade (with 8 bacteria per host species). Larsen et al. [1] then obtained the average number of unique bacterial species per insect host across these three genera (10.7 bacterial species), and then used this number in their subsequent projections of bacterial diversity.

Louca et al. [4] pointed out that Larsen et al. [1] merely estimated the number of unique bacterial species per insect host species. This is correct: Larsen et al. [1] did not directly count the number of host-specific bacteria per insect species, which would have been preferable (see Text S3). However, Louca et al. [4] did not directly count them either. Instead, Louca et al. [4] presented their own indirect estimates of insect-associated bacterial diversity using a different method. Their approach involved estimating bacterial species richness across all insect species in each genus. However, they only did this for Cephalotes, and not all three genera examined by Larsen et al. [1]. The exclusion of the other two genera was not explained.

Most importantly, the calculations of Louca et al. [4] were incorrect, and this is obvious from simply counting the number of bacterial species in Cephalotes. Specifically, Louca et al. [4] incorrectly assumed that the estimates of Larsen et al. [1] were drawn randomly from among all 130 species of Cephalotes. In fact, they were only for 7 closely related species in two clades, as noted above and described explicitly by Larsen et al. [1]. As a result of this incorrrect assumption, Louca et al. [4] estimated that there were only 40.1 unique bacterial species in total among all 130 species of Cephalotes. If they had simply counted the number of unique bacterial species among the 7 ant species considered by Larsen et al. [1], they would have seen that there were actually 54 bacterial species among just these 7 ant species (but excluding rare bacterial species, see below). Thus, their estimate of a total of 40.1 bacterial species for the entire genus of 130 ant species was clearly wrong. Indeed, the estimates of Larsen et al. [1] were based on Sanders et al. [5].

I examined the data analyzed by Sanders et al. [5], which those authors made publicly available on Dryad. Using the standard 97% cut-off for distinct bacterial species with 16S (also used by Louca et al. [4]) and including all species, there were a total of 616 bacterial species among just the 25 Cephalotes species sampled. Among these 616 species, relatively few species were shared with the three other closely related ant genera that they sampled (only 77). Among the 539 species found only in Cephalotes, the majority (369) were found in a single species. Thus, their estimate of only 40.1 bacterial species among all 130 species of Cephalotes was grossly incorrect.

**References for S1 Text**

1. Larsen BB, Miller EC, Rhodes MK, Wiens JJ. Inordinate fondness multiplied and redistributed: the number of species on Earth and the new Pie of Life. Quart Rev Biol. 2017;92: 229–265.

2. Colman DR, Toolson EC, Takacs-Vesbach CD. Do diet and taxonomy influence insect gut bacterial communities? Mol Ecol. 2012; 21: 5124–5137.

3. Yun J-H, Roh SW, Whon TW, Jung M-J, Kim M-S, Park D-S, et al. Insect gut bacterial diversity determined by environmental habitat, diet, developmental stage, and phylogeny of host. Appl Environ Microbiol. 2014; 80: 5254–5264.

4. Louca S, Mazel F, Doebeli M, Parfrey LW. A census-based estimate of Earth’s bacterial and archaeal diversity. PLoS Biol. 2019; 17: e3000106.

5. Sanders JG, Powell S, Kronauer DJC, Vasconcelos HL, Frederickson ME, Pierce NE. Stability and phylogenetic correlation in gut microbiota: lessons from ants and apes. Mol Ecol. 2014; 23: 1268–1283.
