# Supplementary material for: Vast (but avoidable) underestimation of global biodiversity
Source: PLoS Biol. 2021 Aug 12;19(8):e3001192. doi: 10.1371/journal.pbio.3001192 (PMC8360379; doi:10.1371/journal.pbio.3001192)
Supplement: S2 Text — (DOC) [file pbio.3001192.s002.doc]

**S2 Text. Overlap of bacterial species among host animal genera**

Louca et al. [1] claimed that “it is known that substantial overlap exists between the microbiota of different host genera and even of distantly related animal taxa.” They provided no quantitative evidence to support this strong assertion. They cited some studies that they suggested supported this assumption [2–5]. However, they did not provide any numbers from these studies that would support the idea of “substantial overlap.”

The cited studies do not actually support this assumption. For example, they cited a study on fish bacteria as evidence that bacterial species are shared among host species in different genera [3]. That study did not directly show such a pattern. Sullam et al. [3] did directly show that different bacterial higher taxa (e.g. phyla, orders) are shared among fish genera, but that says nothing about whether any bacterial *species* are actually shared among host genera, and (more importantly) to what extent. Even if many bacterial species were shared among host genera (which was not actually shown), this does not contradict the idea that many other bacterial species are unique to each host species (which was the focus of Larsen et al. [6]). Furthermore, fish are not insects. This matters because fish represent a relatively small slice of animal diversity (~32,000 species out of out of ~1.3 million described species; [7]), whereas insects make up the majority (~940,000; [7]), even when considering only described species.

Louca et al. [1] also cited a study on mammalian microbiotas [2], but this seems even more irrelevant to species diversity of bacteria in insects and across animals in general. Yet, that study also rejects their main conclusion: it shows that most sampled bacterial species are not broadly distributed among the sampled host species, and almost all are confined to only one or a few host species (see Fig. 2 of [2]).

Considering this numerical dominance of insect species, the most relevant case studies are from insects. Louca et al. [1] cited a paper by Salzman et al. [5] as evidence for sharing of bacterial species among insect host genera. However, Louca et al. [1] provided no numbers from that study. Salzman et al. [5] found that among five sampled insect species in five genera (two lepidopterans, three beetles) that feed on cycads, there were five bacterial species that were shared among all five genera and occurred at high abundance in all species. Although this might sound impressive in isolation, that study [5] actually reported finding 1,789 bacterial species among these five genera (substantial filtering by abundance reduced the dataset to only 177 species). This makes the sharing of five bacterial species among these five insect genera seem a bit underwhelming, to say the least. Yet, another way of thinking of the conclusions of that study [5] is that even when different insect genera are feeding on the same host plant group, only a fraction of their bacterial species are shared among them. Indeed, Salzman et al. [5] concluded that these five species were “generally distinct in overall bacterial community composition” and that despite some sharing of bacterial species among two sampled species: “each insect harbours a distinctive species-specific bacterial assemblage” (p. 733). This is basically the opposite of what Louca et al. [1] implied that this study showed. Note also that four of the five insect genera were sampled from the same cycad genus (*Zamia*) in the same geographic location (southern Florida). This hardly justifies assuming that all animal genera can share bacterial species, regardless of their phylogeny, habitat, host plant, or geographic range. Furthermore, it seems disingenuous to use a study that found 1,789 bacterial species (or even only 177) among only five sampled insect species to justify assuming that there are only 40,100 bacterial species among all animal species on Earth, without mentioning any relevant numbers from that study.

Louca et al. [1] also cited a study on fungus-farming insects [4] to justify assuming that bacterial species are widely shared among animal genera. However, that study did not provide data on the overall species richness of bacteria among the sampled species (nor on the distribution of all the sampled bacterial species among the sampled insect species). Therefore, the actual prevalence of sharing of bacterial species among insect species and genera in that study is unclear. Thus, there is no basis for claiming that this study shows that “substantial overlap exists between the microbiota of different host genera” as claimed by Louca et al. [1]. Again, the fact that some bacterial species are shared among some insect genera does not contradict the idea that each insect species harbors many other bacterial species that are specific to the host species, and the relevant numbers were not presented by Aylward et al. [4] nor by Louca et al. [1].

What is very surprising is that Louca et al. [1] cited these particular studies to justify their assumption that all bacterial species can be shared among all animal genera. In some ways, these studies showed the opposite: they seem to show a limited number of bacterial species being shared among insect genera under unusual circumstances (e.g. shared use of cycads, shared use of fungus-farming for food).

Finally, even though some studies found some sharing of bacterial species among more distantly related insects, other analyses show that bacterial microbiotas within insect species are significantly correlated with the phylogeny of their hosts. For example, Sanders et al. [8] used data from *Cephalotes* ants (and related genera) to show that microbial communities within species tend to cluster based on host phylogeny, with the microbiotas of different genera being the most distinct. Again, Louca et al. [1] based all their estimates of animal microbial diversity on this genus. Similarly, Brucker and Bordenstein [9] found that in *Nasonia*, the similarity of bacterial communities among insect host species is related to the host’s phylogeny. A broad study across 218 insect species and 21 orders [10] also found that insect bacterial microbiotas were related to both insect diet and to host phylogeny. In summary, these results strongly suggest that it would be highly inappropriate to assume (as done by Louca et al. [1]) that any randomly selected pair of animal genera can share bacterial species, regardless of their phylogeny, habitat, geographic range, or diet.

**References for S2 Text**

1. Louca S, Mazel F, Doebeli M, Parfrey LW. A census-based estimate of Earth’s bacterial and archaeal diversity. PLoS Biol. 2019; 17: e3000106.

2. Muegge BD, Kuczynski J, Knights D, Clemente JC, González A, Fontana L, et al. Diet drives convergence in gut microbiome functions across mammalian phylogeny and within humans. Science. 2011; 332:970–974.

3. Sullam KE, Essinger SD, Lozupone CA, O’Connor MP, Rosen GL, Knight R, et al. Environmental and ecological factors that shape the gut bacterial communities of fish: a meta-analysis. Mol Ecol. 2012; 21:3363–3378.

4. Aylward FO, Suen G, Biedermann PHW, Adams AS, Scott JJ, Malfatti SA, et al. Convergent bacterial microbiotas in the fungal agricultural systems of insects. mBio. 2014; 5:e02077.

5. Salzman S, Whitaker M, Pierce NE. Cycad-feeding insects share a core gut microbiome. Biol J Linn Soc. 2018; 123: 728–738.

6. Larsen BB, Miller EC, Rhodes MK, Wiens JJ. Inordinate fondness multiplied and redistributed: the number of species on Earth and the new Pie of Life. Quart Rev Biol. 2017;92: 229–265.

7. Roskov Y. et al. 2014. Species 2000 and ITIS Catalogue of Life, 29 October 2014. Leiden (The Netherlands): Species 2000, 2014. Available at http://www.catalogueoflife.org/col. Accessed 20 December 2020.

8. Sanders JG, Powell S, Kronauer DJC, Vasconcelos HL, Frederickson ME, Pierce NE. Stability and phylogenetic correlation in gut microbiota: lessons from ants and apes. Mol Ecol. 2014; 23: 1268–1283.

9. Brucker RM, Bordenstein SR. The roles of host evolutionary relationships (Genus: *Nasonia*) and development in structuring microbial communities. Evolution 2012;66: 349–362.

10. Yun J-H, Roh SW, Whon TW, Jung M-J, Kim M-S, Park D-S, et al. Insect gut bacterial diversity determined by environmental habitat, diet, developmental stage, and phylogeny of host. Appl Environ Microbiol. 2014; 80: 5254–5264.
