# Supplementary material for: Vast (but avoidable) underestimation of global biodiversity
Source: PLoS Biol. 2021 Aug 12;19(8):e3001192. doi: 10.1371/journal.pbio.3001192 (PMC8360379; doi:10.1371/journal.pbio.3001192)
Supplement: S3 Text — (DOC) [file pbio.3001192.s003.doc]

**S3 Text. Refining the estimates of Larsen et al. (2017)**

Given that the estimates of Louca et al. [1] are problematic, what should the correct numbers be? Here, I will first briefly revisit the calculations of Larsen et al. [2], using more direct estimates of host-specific bacterial richness for each insect genus. I will then provide overall re-estimates of global biodiversity.

First, among the 17 species of Drosophila (data from [3]) simply counting the number of bacterial species unique to each insect host species yields 6.4 host-specific bacterial species per insect species (but note that this includes two species with data from the lab only, which have only 0 and 1 unique bacterial species, respectively). This is lower than the 13.4 initially estimated by Larsen et al. [2], but is more similar to the estimates for the other two genera.

For Cephalotes, the original estimates of Larsen et al. [2] considered only the ant species in the laminatus and depressus species groups (4 and 3 species respectively; data from [4]). They also ignored bacterial species found in only one individual of a given insect host species. Including these bacterial species could either increase or decrease the number of bacterial species that are considered unique to an insect host species (therefore it is not necessarily more conservative to exclude them when estimating the number of bacterial species unique to each host species). These species counts were therefore recalculated after including these additional bacterial species, and looking across all sampled Cephalotes and related genera to ensure that bacterial species were indeed host-species specific (given the available sampling). This yielded a mean of 7.1 unique bacterial species per insect host species, and 84 bacterial species in total among just these 7 Cephalotes species. Note that other estimates for Cephalotes and related genera also include all bacterial species (i.e. 1,019 among the four genera, 616 in *Cephalotes*), regardless of how widespread they were among individuals within each species.

Third, for Nasonia [5], direct counts of unique bacterial species in each insect host species were not available: this is why Larsen et al. [2] used an indirect method for estimating bacterial richness for all three genera (rather than direct counts). However, it is possible to use Fig. 2 of Brucker and Bordenstein [5] to directly count all the bacterial higher taxa (genus, family) that were present in only one wasp species as a unique species. This could underestimate the number of unique species because there could be different bacterial species in different host species that are in the same bacterial family or genus. Nevertheless, using this approach yields an estimate of mean bacterial species unique to each wasp species of 9.3. This is almost identical to the original estimate of 9.6 from Larsen et al. [2].

Given these results for each genus, I then re-estimated the mean bacterial species richness per insect host species based on these genera (Drosophila=6.4; Cephalotes=7.1; Nasonia=9.3). This yielded a value of 7.6 unique bacterial species per insect host species. This is different from the estimate of 10.7 by Larsen et al. [2], but still similar overall. In Table 2 of the main text, I present estimates of global biodiversity based on these new estimates, following the methods of Larsen et al. [2]. These new estimates are generally within the range of values estimated in that study. Specifically, Larsen et al. [2] projected 0.209–5.8 billion species on Earth, of which 66–91% are bacteria, whereas I project here that there are 0.183–4.2 billion species, of which 58–88% are bacteria (Table 2).

It is important to note that the number of bacteria species unique to each insect host provides only a minimum estimate of insect-associated bacterial species richness. These counts ignore bacterial species that are shared by two or more host species. Indeed, many bacterial species in these three insect host genera occur in more than one species, but in only a subset of the sampled species in that genus. Similarly, there could be many species that are specific to each genus (or shared among closely related genera) that would be completely ignored by this approach.

Note also that these estimates assume that bacterial species that appear to be unique to a single host species are not instead shared among genera. This assumption is supported by detailed studies showing that sharing of bacterial species among host species is related to host phylogeny [4–6]. Therefore, we should expect to see the most sharing of bacterial species among the most closely related host species (e.g. in the same genus). Recall that in Cephalotes [4], most bacterial species found in this genus were not shared with related genera (88%; 539/616), and most sampled species in the genus appear to be species-specific, even when other genera are included in the assessment (68%; 369/539). Importantly, the sharing of bacterial species with other sampled ant genera (77 species shared) seems to be much smaller than the underestimation of richness associated with considering only host species-specific bacterial species (170 bacterial species were found in more than one *Cephalotes* species, but only in *Cephalotes* and not in related ant genera).

Finally, I recognize that the set of approaches used here to estimate global biodiversity has many assumptions. These are addressed at length in Larsen et al. [2], and many are also made by Louca et al. [1]. These include the use of the standard 97% divergence cutoff in the 16S gene to recognize distinct bacterial species (which may underestimate or overestimate true bacterial species richness), and whether bacterial species are comparable to eukaryotic species. Other researchers might prefer to ignore relatively uncommon bacterial species (although this preference seems problematic for estimating overall global biodiversity). I have also not included free-living bacterial species in these estimates, given the assumption that their diversity will be minor relative to host-associated bacteria (note that the low diversity of free-living bacteria was the major conclusion of Louca et al. [1]). Moreover, higher richness of free-living bacteria would not lead to lower estimates of global biodiversity and would still support bacterial domination of the Pie of Life. I also recognize that I have not systematically searched the literature for additional case studies of bacterial species among closely related insect species. My main goal here is to address the problematic estimates of Louca et al. [1], who based their global-scale estimates on one genus only. It would be very strange to dismiss my re-analysis here because it includes only three genera, in favor of a re-analysis that included only one of them [1].

**References for S3 Text**

1. Louca S, Mazel F, Doebeli M, Parfrey LW. A census-based estimate of Earth’s bacterial and archaeal diversity. PLoS Biol. 2019; 17: e3000106.

2. Larsen BB, Miller EC, Rhodes MK, Wiens JJ. Inordinate fondness multiplied and redistributed: the number of species on Earth and the new Pie of Life. Quart Rev Biol. 2017;92: 229–265.

3. Chandler JA, Morgan Lang J, Bhatnagar S, Eisen JA, Kopp A. Bacterial communities of diverse *Drosophila* species: ecological context of a host-microbe model system. PLOS Genetics 2011;7: e1002272.

4. Sanders JG, Powell S, Kronauer DJC, Vasconcelos HL, Frederickson ME, Pierce NE. Stability and phylogenetic correlation in gut microbiota: lessons from ants and apes. Mol Ecol. 2014; 23: 1268–1283.

5. Brucker RM, Bordenstein SR. The roles of host evolutionary relationships (Genus: *Nasonia*) and development in structuring microbial communities. Evolution 2012;66: 349–362.

6. Yun J-H, Roh SW, Whon TW, Jung M-J, Kim M-S, Park D-S, et al. Insect gut bacterial diversity determined by environmental habitat, diet, developmental stage, and phylogeny of host. Appl Environ Microbiol. 2014; 80: 5254–5264.
